# Anxiety and Depression Among College Students Before and After the COVID‐19 Pandemic Lockdown Lift: A Network Analysis Study Focus on the Transition Period

**DOI:** 10.1002/pchj.70028

**Published:** 2025-06-26

**Authors:** Jia‐li Liu, Wan‐ting Ran, Zhi Wang, Ze‐min Nie, Gui‐lin Huang, Jun‐wen Yi, Si‐yu Yang, Zi‐yi He, Ya Wang, Gui‐fang Chen

**Affiliations:** ^1^ Affiliated Hospital of Zunyi Medical University Zuiyi China; ^2^ Zunyi Medical University Zunyi China; ^3^ School of Psychology Capital Normal University Beijing China; ^4^ Neuropsychology and Applied Cognitive Neuroscience Laboratory, CAS Key Laboratory of Mental Health Institute of Psychology Beijing China; ^5^ Department of Psychology University of Chinese Academy of Sciences Beijing China

**Keywords:** anxiety, COVID‐19 epidemic, cross‐lagged panel network analysis, depression, network analysis

## Abstract

Since the outbreak of the COVID‐19 pandemic, college students experienced changed campus life during the evolving pandemic restrictions. Anxiety and depression have become increasingly prevalent, leading to the necessity for further examining their relationship and comorbidity. This study used the network analysis to investigate the interaction and causal relationship in the anxiety‐depression network among Chinese college students during the pandemic. A longitudinal survey with two specific points among 705 college students were conducted from 12 December to 30 December 2022 (lockdown period, T1), and from 8 February to 13 March 2023 (lockdown lift period, T2). Contemporaneous network and cross‐lagged panel network (CLPN) analysis were conducted to examine the issue from both cross‐sectional and longitudinal perspectives. Both contemporaneous networks exhibited extensive links between anxiety and depression symptoms. The key central symptom was “STAI16: [Not] content” at T1, and was “STAI15: [Not] relaxed” at T2. CLPN analysis suggested that “STAI15: [Not] relaxed” had the highest in‐prediction, while “STAI13: Jittery” had the highest out‐prediction. The strongest transdiagnostic prediction was from “BDI6: Punishment” to “STAI9: Frightened”, and the bridge symptoms in both contemporaneous networks and CLPN included overlaps like “STAI11: [Not] self‐confident” and “STAI14: Indecisive”, which served as important symptoms contributing to anxiety‐depression comorbidity. These findings provide new insights into the causal relationships between depression and anxiety before and after lockdown lift, shed light on the comorbidity factors, and provide support for targeted interventions to address mental health challenges faced by college students in public crisis.

## 1 Introduction

Since the coronavirus disease 2019 (COVID‐19) outbreak around the world, the COVID‐19 pandemic has escalated into a global health emergency. The widespread epidemic and long‐term sequelae not only harm physical health, but also cause lasting mental health issues, such as a surge in the prevalence of depression and anxiety (Santomauro et al. 2021; World Health Organization 2022). Previous studies showed a higher prevalence of anxiety and depression during the COVID‐19 pandemic in Chinese people (Wang et al. 2020; Bin et al. 2024), and a meta‐analysis showed that the overall prevalence of depression and anxiety among Chinese college students was 27.0% and 26.0% during the COVID‐19 pandemic, respectively (Zhang et al. 2021). To prevent the spread of pandemics, a variety of measures, including mass lockdowns and school closures, have disrupted the daily routines of college students. Lockdowns are two sides of the same coin: they help control the spread of the pandemic and protect students from infection (Ren 2020), while also lead to a series of negative effects on mental health among college students (Huang and Liu 2023; Xu et al. 2022; Essadek et al. 2022; Elmer et al. 2020). College students face heightened emotional risks due to reduced parental support (Lowe and Dotterer 2018), and the prevalence of one or more mental health problems increased nearly 50% from 2013 to 2021 (Lipson et al. 2022). They have to deal with the academic load and the challenges of career planning (Pedrelli et al. 2015), and are more prone to experiencing mental health issues compared to many other subpopulations (Auerbach et al. 2018). The campus lockdowns have disrupted the campus study and work routine, interfered with the convenience of campus life, and caused problems in work and leisure (Gloster et al. 2020), leading to a high prevalence of anxiety, depression, and even their comorbidity (Zhang et al. 2021; Xu et al. 2022).

Depression and anxiety are characterized by overlapping pathogenic and psychological factors, including emotional regulation difficulties, neuroticism, and excessive responses to unwanted emotions (Curtiss and Klemanski 2016; Mennin and Fresco 2015), and exhibit a high rate of comorbidity worldwide (McGrath et al. 2020). Depression and anxiety are reciprocal risk factors for each other (Jacobson and Newman 2017). For example, individuals with anxiety are more vulnerable to depressive symptoms (Möller et al. 2016), possibly due to alterations in cognitive and behavioral patterns that can contribute to depressive episodes (Wittchen et al. 2000). In addition, individuals with comorbidity of anxiety and depression tend to demonstrate more pronounced symptomatology, greater impairment in social functioning, and severer treatment refractory compared with those with isolated disorders (Saha et al. 2021; Belzer and Schneier 2004). A review showed that the pooled prevalence of comorbid depression and anxiety among college students in the United States was 20.7% between 2013 and 2019, with a twofold increase from 11.9% to 24.4% during this timeframe (Hoeflich et al. 2023). An online cross‐sectional survey suggested that the prevalence of comorbid depression and anxiety was 25.60% among Chinese college students during the COVID‐19 lockdown (Huang and Liu 2023). Therefore, it was of great significance to further examine depression, anxiety, and their comorbidity in college students, especially during the critical time of the pandemic. However, conventional research methodologies typically assess symptom severity and comorbidity by evaluating the aggregate scores of scales, while symptoms are secondary to an underlying common cause (Schmittmann et al. 2013), which overlook the interactions and inter‐relationships between individual symptoms.

To characterize the interactions between depression and anxiety symptoms, network analysis, an emerging novel statistical method, can specify dynamic and reciprocal relationships between comorbid disorders and identify targets for treatment (Robinaugh et al. 2020). It can be used to identify central symptoms with stronger inter‐symptom connections (Epskamp and Fried 2018), as well as bridge symptoms connecting the two mental disorders as pathways (Jones et al. 2021), which are believed to drive the coexistence and comorbidity of various disorders (Afzali et al. 2017; Qi et al. 2021). A systematic review of the network model of depressive and anxiety symptoms has suggested that “Sad mood” and “Uncontrollable worry” served as the most common central symptoms, while “Sad mood” and “Restlessness” played an important bridging role in comorbidity (Cai et al. 2024). Furthermore, previous studies have suggested that motor‐related symptoms in depression are closely related to anxiety symptoms (Bai, Cai, et al. 2021; Bai, Xi, et al. 2021; Cai et al. 2022, 2024). During the COVID‐19 pandemic period, there are also numerous studies applying network analyses to examine anxiety and depressive symptom relations and uncover the models underlying the pathway to comorbidity (Li et al. 2024; Cai et al. 2022; Wang et al. 2020). Specifically, for Chinese college students, “Fatigue”, “Uncontrollable worry”, and “Excessive worry” are the most commonly mentioned central symptoms, and “Restlessness” may be key bridge symptoms that contribute to the comorbidity across the pandemic stages (Tao et al. 2024; Bai, Cai, et al. 2021; Bai, Xi, et al. 2021; Li et al. 2024).

However, the majority of network studies on depressive and anxiety symptoms during the COVID‐19 pandemic have been cross‐sectional studies depicting associations at a single time point (Bai, Cai, et al. 2021; Bai, Xi, et al. 2021; Tao et al. 2024), or trend studies observing overall trends from different samples at multiple time points (Liu et al. 2022; Wang et al. 2020). Such studies can illustrate the connection between the two symptoms at specific time points, but cannot indicate the causal effect. The structure of the depression‐anxiety network undergoes alterations, and comorbidity temporally fluctuates across different stages of the pandemic (Zavlis et al. 2021; Liu et al. 2022). For example, “Restlessness” was the central and bridge symptom during the COVID‐19 outbreak, whereas after the peak, “Irritability” became the central and bridge symptom in Chinese participants (Y. Wang et al. 2020), and the expected influence of “Restlessness” increased slowly during the lockdown lift period in Chinese college students (J. Li et al. 2024). To elucidate the dynamic associations in symptom‐symptom interaction during the course of the pandemic, a CLPN would be a novel and helpful method, which integrates latent variable models and network analysis to discover longitudinal processes over time (Wysocki et al. 2022). A study investigating the CLPN of anxiety and depressive symptoms in England older adults found that “Nervousness” was the one most affected by other symptoms, and “Restless sleep” was the one that most affected other symptoms from the first wave to the second wave of the pandemic (Ramos‐Vera et al. 2024). Nevertheless, no research to date has explored the causal relationship in the anxiety‐depression network before and after lifting the pandemic lockdown in Chinese college students.

To address these gaps, we first examined the contemporaneous networks of comorbidity between depression and anxiety among Chinese college students during the COVID‐19 lockdown period and after the lockdown lift to elucidate the causes of comorbidity. Then we used CLPN to explore the cross‐lagged effect between variables and assess the causal relationships between depression and anxiety during the two distinct periods of the pandemic. This study aimed to identify central and bridge symptoms and present the strongest symptom‐to‐symptom interactions, providing theoretical support for precise targeted interventions to address the mental health challenges faced by college students during the public health and collective trauma events.

## 2 Methods

### 2.1 Study Design and Participants

A longitudinal survey with two time points in college students was conducted in Zunyi, Guizhou province, China. The first survey was conducted from December 12 to December 30, 2022 (lockdown period, T1). At this point, while some regions in China have announced adjustments to their pandemic response policies, such as Shanghai, strict prevention and lockdown measure were still implemented in Guizhou province. College students were asked to remain on campus, unless there was an urgency. The second survey was conducted from February 8 to March 13, 2023 (lockdown lift period, T2), 2 months since T1. After the widespread of the COVID‐19 pandemic outbroke and then subsided, pandemic control policies changed during this period, including the cessation of routine nucleic acid testing, designated quarantine protocols, and fully lifted lockdown. College students were no longer restricted to campus and returned to normal lives, although a lot of students either contracted or were still recovering from COVID‐19 infections in this phase.

The data were collected through an online platform (www.wjx.cn), and the college students in Zunyi were recruited. The inclusion criteria were: (1) college students between the ages of 18 and 25; (2) residing in China during the COVID‐19 outbreak. All participants provided informed consent before the online survey. Finally, a total of 705 college students completed both surveys (T1: *n* = 995, T2: *n* = 869). The study was approved by the Biomedical Ethics Committee of the Affiliated Hospital of Zunyi Medical University (KLL‐2022‐449).

### 2.2 Measures

#### 2.2.1 State Anxiety

The State–Trait Anxiety Inventory (STAI) is a self‐report scale to evaluate anxiety levels (Spielberger et al. 1980) with good reliability and validity in Chinese samples (Shek 1988). The 20‐item State‐STAI is a subscale used to measure state anxiety, scored using a Likert‐type scale ranging from 1 to 4, with reverse scoring (5—raw score) applied to 10 of the items. A higher level of score indicates greater anxiety. The Chinese version of STAI showed high reliability at T1 (Cronbach's *α* = 0.909) and T2 (Cronbach's *α* = 0.902) in the study. A score of 40 is the cutoff point for state anxiety (Spielberger et al. 1980).

#### 2.2.2 Depression

The Beck Depression Inventory (BDI) is a 21‐item questionnaire to assess the level of depressive symptoms (Beck et al. 1988). Each item has four statements and participants need to select one that is most suitable to them. The score of each item ranged from 0 to 3. Higher scores indicate the existence of higher levels of depression. The Chinese version of the BDI has good reliability and validity (Shek 1990). The Cronbach's alpha coefficients of BDI were 0.917 at T1 and 0.909 at T2. A score of 14 is the cutoff point for moderate depression (Wang et al. 1999).

### 2.3 Data Analysis

#### 2.3.1 Descriptive Analysis

The mean and standard deviation of age, STAI, and BDI were calculated. Paired samples *t*‐tests were performed to assess differences in STAI and BDI between T1 and T2. All analyses were conducted via SPSS v24.0. The significance level was set at *p* < 0.05.

#### 2.3.2 Contemporaneous Network Analysis

All network analyses were conducted via R 4.3.1 statistical software (R Team 2014). The relationships between anxiety and depression at the lockdown period and at lockdown lift period were examined. In particular, the reversed scores of the 10 items of STAI were applied rather than the raw scores to maintain consistent expression, where higher scores indicate more severe anxiety symptoms. Items with reversed scores are presented by inserting [Not] into the item, and item descriptions of STAI and BDI were simplified to enhance readability. For example, “STAI1: [Not] calm”. Two contemporaneous networks were constructed using the Extended Bayesian Information Criterion Graphical Least Absolute Shrinkage and Selection Operator (EBICglasso) method, and the tuning parameter was set as 0.5 and applied to shrink the partial correlation coefficients for a more stable and interpretable network (Friedman et al. 2008).

To assess the importance of each node and illuminate how nodes are interconnected in the network, strength was chosen as the key index for central symptoms (Opsahl et al. 2010), as this index is the most commonly used measure of depression–anxiety networks in previous research (Cai et al. 2024). Network visualization of the networks was performed with “qgraph” package (Epskamp et al. 2012), within which nodes indicated symptoms and edges represented partial correlations between nodes. The threshold of edge weight was set at 0.05, and low‐weight edges were removed to retain the strongest edges, and “bootnet” package (Epskamp and Fried 2018) was used to analyze the accuracy and stability of the estimated networks by 3000 non‐parametric bootstrapping and 3000 case‐drop bootstrapping.

In addition, to identify the symptoms linking pathway between depression and anxiety symptoms, the bridge centrality index (e.g., bridge strength) was estimated via the applied bridge function in the “networktools” package (Jones 2017). In the study, nodes identified as bridge nodes at T1 and T2 were those ranked in the top 10% for bridge strength, which was calculated as the sum of the edges between a node and all nodes from different communities (Jones et al. 2021).

#### 2.3.3 CLPN Analysis

The CLPN was estimated from T1 to T2 assessment by using the “glmnet” package (Friedman et al. 2010). Based on the guidance of CLPN to increase interpretability (Wysocki et al. 2022), the LASSO regression with 10‐fold cross‐validation parameter selection was used to estimate the CLPN for the causal relationships of anxiety and depression among college students (Wysocki et al. 2022). Consistent with the literature (Li and Kwok 2023), autoregressive pathways for each node estimated by CLPN were not included when calculating the values of centrality and prediction to focus on the causal relationships between different symptoms. The threshold of edge weight is set at 0.05, and low‐weight edges were removed. The results were then summarized, and plots produced with “qgraph” package (Epskamp et al. 2012).

Besides centrality indexes, cross‐lagged in‐prediction and out‐prediction are also important indexes about nodes of the CLPN. Higher in‐prediction values indicate that the node is more easily influenced by other nodes at the previous time point, while higher out‐prediction values indicate that the nodes exerted more significant influences on all other nodes at the later time point. In addition, to figure out the core bridge symptom and pathway to comorbidity of the CLPN, bridge in‐prediction (cross‐construct in‐prediction, indicating how much each variable is predicted by variables belonging to other construct), and bridge out‐prediction (cross‐construct out‐prediction, indicating how much each variable predicts variables belonging to other construct) were also included in the analysis. It is important to note that, even though in‐prediction and out‐prediction values can range from 0 to 1, out‐prediction is typically substantially lower than in‐prediction (Wysocki et al. 2022). Therefore, in this study, we multiplied the out‐prediction by 100 for better graphical representation and description. See more detailed calculation methods and codes on the Psychological Science Data Center (https://www.scidb.cn/s/63Ibqu). Similarly, the CLPN accuracy and stability was analyzed by 3000 nonparametric type and 3000 case‐drop type bootstraps (Epskamp and Fried 2018).

## 3 Results

### 3.1 Descriptive Statistics

The demographic characteristics of all participants are presented in Table 1. The kurtosis and skewness statistics of STAI and BDI indicated that the data in this study were normally distributed at both T1 and T2. There was no significant difference in age, gender, depression, or anxiety between participants who completed both surveys and those who only completed T1 survey at baseline, although a marginal trend was observed for depression. In T1, 38.3% of college students had depression symptoms (BDI ≥ 14), 55.7% had anxiety symptoms (STAI ≥ 40), and 32.3% had their comorbidity. During T2, 35.0% of college students had depression symptoms, 49.8% had anxiety symptoms, and 27.5% had their comorbidity. And participants showed significantly decreased anxiety (*t*_(704)_ = −2.84, *p* = 0.005) and depression (*t*_(704)_ = −2.35, *p* = 0.019) at T2 compared to T1. See Table S1 for a complete list of all items and mean (SD) scores of STAI and BDI at T1 and T2.

**TABLE 1:** Description of demographic characteristics of participants (*n* = 705) and comparison of anxiety and depression between T1 and T2.

		Completed both surveys		Completed				
Variables	Statistic	T1	T2	T1 vs T2		Only T1	Only T1 VS T2	
		(*n* = 705)		*t*	*p*	(*n* = 290)	*t/x* ^2^	*p*
Age	Mean (SD)	19.14 (0.99)		\	\	19.23 (1.19)	1.26	0.210
Female gender	*N* (%)	552 (78.3%)		\	\	231 (79.7%)	0.23	0.635
STAI (20–80)	Mean (SD)	40.47 (9.12)	39.63 (8.75)	2.84	0.005	41.06 (9.27)	0.92	0.357
	Skewness	0.09	0.04			−0.11		
	Kurtosis	−0.02	−0.20			−0.26		
BDI (0–63)	Mean (SD)	12.05 (8.07)	11.39 (8.64)	2.35	0.019	13.44 (10.67)	1.94	0.053
	Skewness	0.86	0.86			0.78		
	Kurtosis	0.36	0.44			−0.11		

Abbreviations: BDI, Beck depression inventory; SD, standard deviation; STAI, State–Trait Anxiety Inventory.

### 3.2 Contemporaneous Networks

The contemporaneous networks exhibited extensive positive correlations pervading among nodes, both at T1 (128/820 above 0.05) and T2 (128/820 above 0.05). See Figure S1–S4 for more details of the stability and accuracy of the two contemporaneous networks.

**FIGURE 1:**
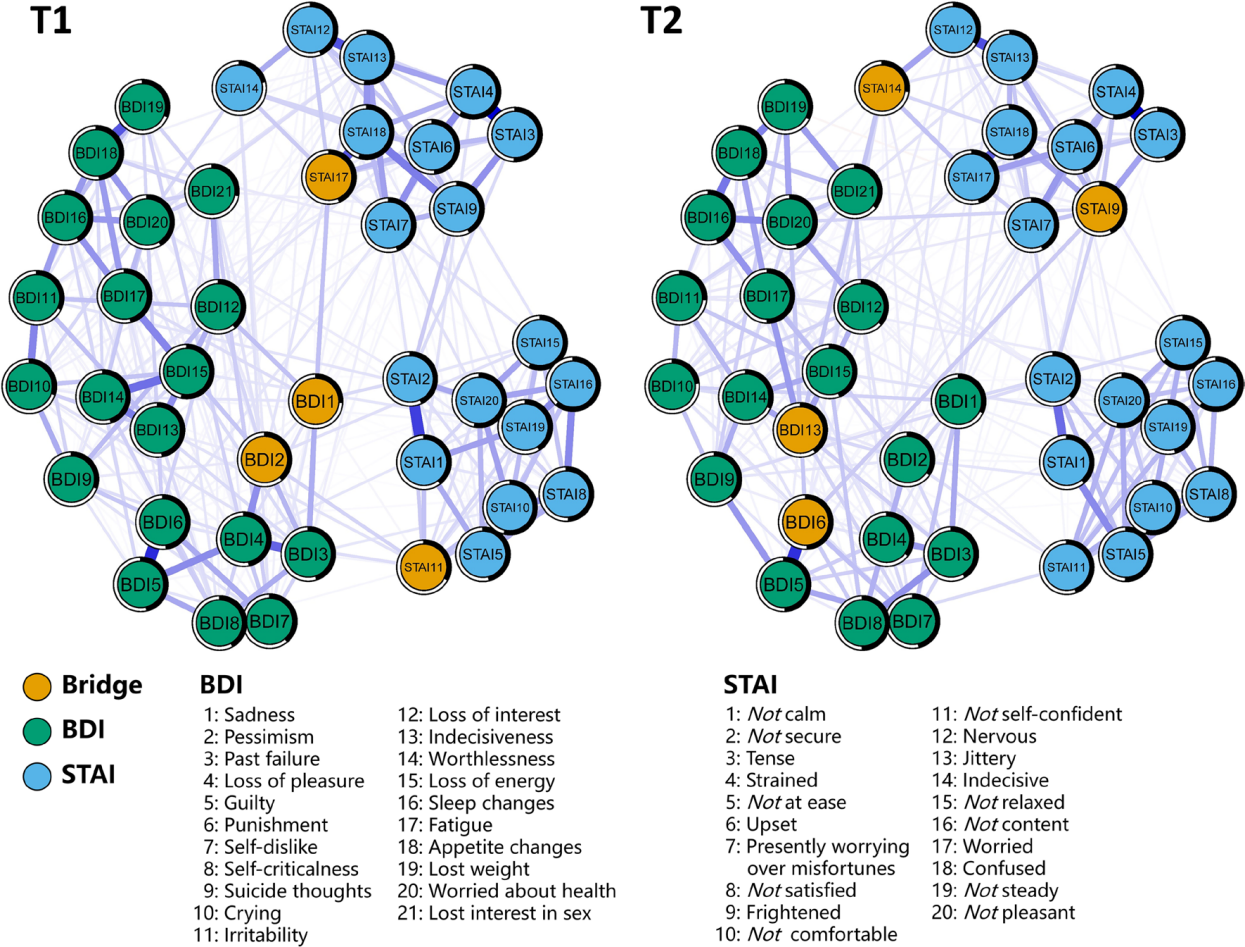
The contemporaneous networks of anxiety‐depression symptoms at T1 and T2. Blue edges indicate positive partial correlations, and red edges indicate negative partial correlations. Thicker lines represent stronger partial correlations. The black ring around each node represents its predictability values. The bridge nodes (yellow nodes) indicate the nodes which ranked at the top 10% for bridge strength. BDI, Beck depression inventory; STAI, State–trait anxiety inventory. The threshold is set at 0.05, and low‐weight edges are removed.

As shown in Figure 1, for the T1 network, the strongest edges were “STAI3: Tense”—“STAI4: Strained” (*r =* 0.38); “STAI15: [Not] relaxed”—“STAI16: [Not] content” (*r =* 0.34); and “BDI5: Guilty feelings”—“BDI6: Punishment” (*r =* 0.32). In addition, the edges exhibited the strongest relation between communities was “STAI17: Worried”—“BDI1: Sadness” (*r =* 0.09). For the T2 network, the three strongest edges were identical to those at T1, with the same order (*r =* 0.41, 0.38, and 0.33). In addition, the edge between communities exhibited the strongest relation was “STAI14: Indecisive”—“BDI13: Indecisiveness” (*r =* 0.09).

Regarding the centrality indexes, the most central nodes at T1 were “STAI16: [Not] content” with the highest strength, followed by “STAI18: Confused”, “STAI4: Strained”, and “STAI20: [Not] pleasant” (strength = 1.00, 0.99, 0.93, and 0.91). On the other hand, the most central nodes at T2 were “STAI15: [Not] relaxed”, followed by “STAI10: [Not] comfortable”, “BDI20: Worried about health”, and “STAI9: Frightened” (strength = 1.07, 0.93, 0.91, and 0.90).

**FIGURE 2:**
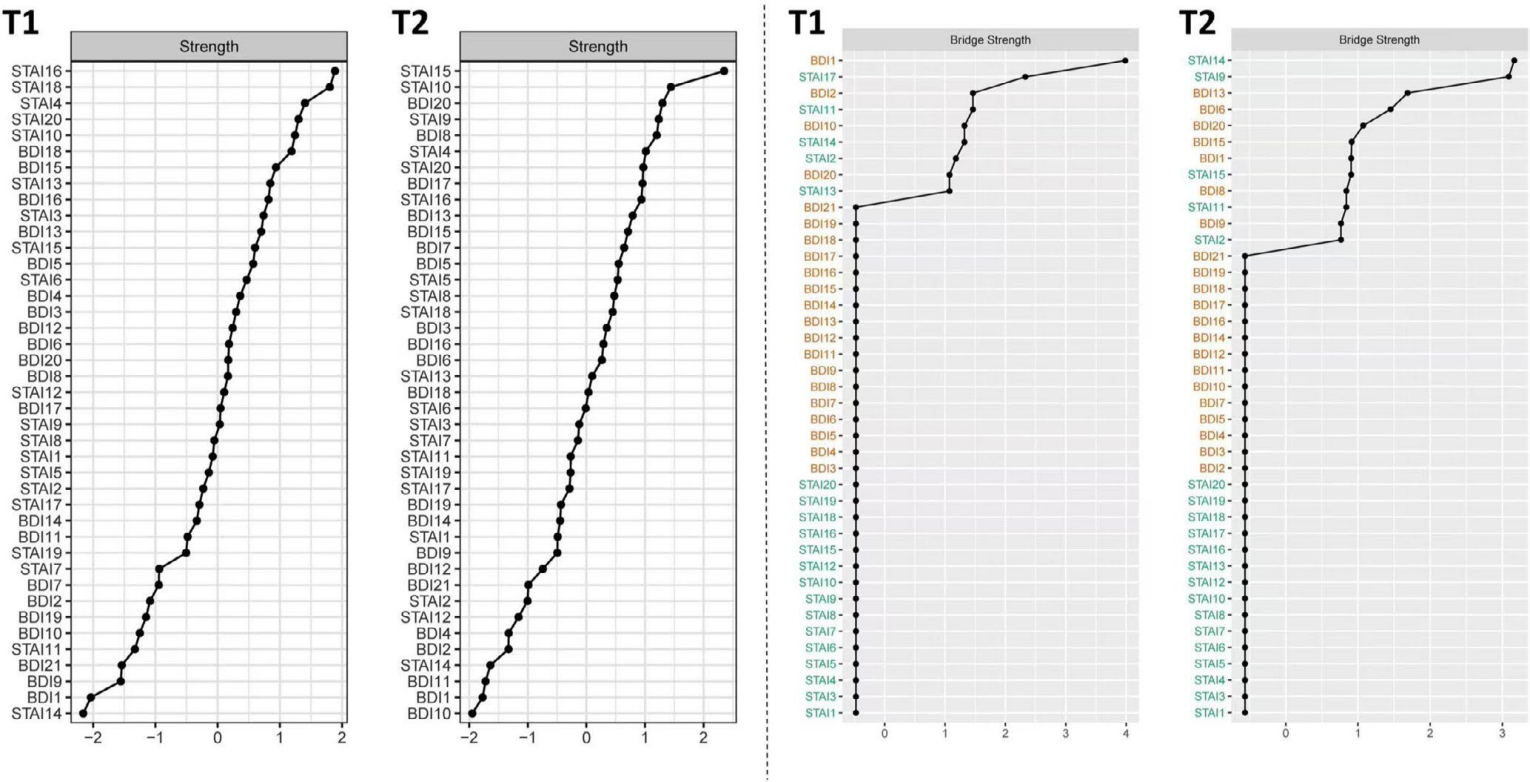
Centrality indexes and bridge centrality indexes of nodes in the contemporaneous networks at T1 and T2. Centrality indexes and bridge centrality indexes are shown as standardized z‐scores. STAI, State–trait anxiety inventory. BDI, Beck depression inventory.

For the bridge symptoms, the bridge nodes at T1 were “BDI1: Sadness”, “STAI17: Worried”, “STAI11: I [Not] self‐confident”, and “BDI2: Pessimism” (Bridge strength = 0.15, 0.09, 0.06, and 0.06). Meanwhile, the bridge nodes at T2 were “STAI14: Indecisive”, “STAI9: Frightened”, “BDI13: Indecisiveness” and “BDI6: Punishment” (Bridge strength = 0.16, 0.15, 0.09, and 0.08). See Figure 2 for more details of the two contemporaneous networks at T1 and T2.

### 3.3 CLPN Analysis

**FIGURE 3:**
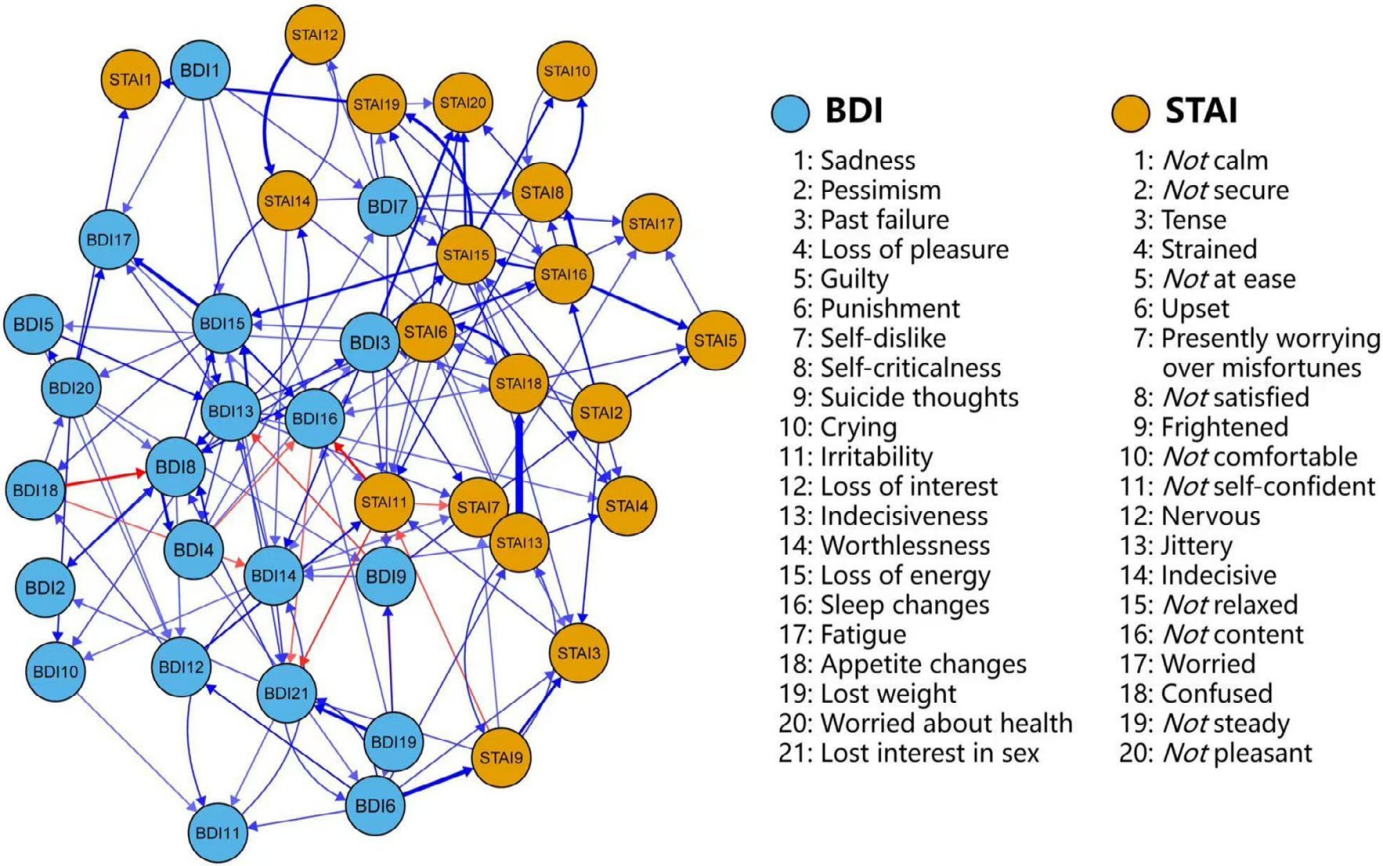
Cross‐lagged panel network of anxiety‐depression symptoms from T1 to T2. The arrows indicate the temporal association between the nodes. Blue edges indicate positive predictions, and red edges indicate negative predictions. Thicker lines represent stronger partial predictions. STAI, State–trait anxiety inventory; STAI, State–trait anxiety inventory. The threshold was set at 0.05 and low‐weight edges were removed.

Structure of the CLPN from T1 to T2 is shown in Figure 3, in which the standardized regressions between the nodes can be observed. The CLPN exhibited extensive positive predictions pervading among nodes (141/1640 above 0.05), while the quantity of negative predictions was relatively rare (10/1640 below −0.05). See Figures S5 and S6 for more details of stability and accuracy of the CLPN.

The edge from “STAI13: Jittery” to “STAI7: Presently worrying over misfortunes” displayed the strongest positive cross‐lagged connection in the CLPN (*β* = 0.18), followed by the positive prediction from “STAI13: Jittery” to “STAI18: Confused”; and from “BDI6: Punishment” to “STAI9: Frightened” (*β* = 0.18 and 0.13). Meanwhile, the edges from “BDI18: Appetite changes” to “BDI8: Self‐criticalness” showed the strongest negative association (*β* = −0.11), followed by the negative prediction from “STAI11: [Not] self‐confident” to “BDI16: Sleep changes” and from “STAI11: [Not] self‐confident” to “BDI21: Lost interest in sex” showed the strongest negative predictions (*β* = −0.10 and − 0.07). See Figure 3 for more details about the CLPN from T1 to T2.

**FIGURE 4:**
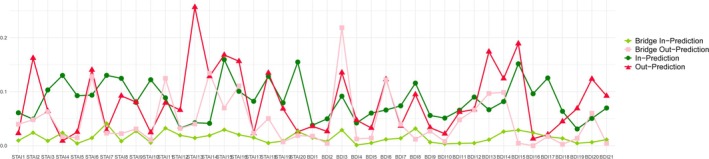
In‐prediction and Out‐prediction of each node in the cross‐lagged panel network from T1 to T2. Because that out‐prediction is typically substantially lower than in‐prediction, the value of (bridge) out‐prediction in the graph is 100 times the original value for the proper rendering of the image. BDI, Beck depression inventory; STAI, State–trait anxiety inventory.

The (bridge) in‐prediction and (bridge) out‐prediction of each node in CLPN from T1 to T2 were calculated and presented in Figure 4. The symptoms with the highest in‐prediction were “STAI15: [Not] relaxed” (in‐prediction = 0.16), followed by “STAI20: [Not] pleasant”, “BDI15: Loss of energy”, and “STAI7: Presently worrying over misfortunes” (in‐prediction = 0.15, 0.15 and 0.13). On the other hand, the nodes with highest out‐prediction were “STAI13: Jittery” (100 * out‐prediction = 0.25), followed by “BDI15: Loss of energy”, “BDI13: Indecisiveness” and “STAI15: [Not] relaxed” (100 * out‐prediction = 0.18, 0.17 and 0.17).

In terms of bridge prediction, the symptoms “STAI7: Presently worrying over misfortunes”, “STAI11: [Not] self‐confident”, “BDI8: Self‐criticalness”, and “STAI15: [Not] relaxed” had the highest bridge in‐prediction (bridge in‐prediction = 0.04, 0.03, 0.03, and 0.03). Correspondingly, the symptoms “BDI3: Past failure”, “STAI14: Indecisive”, “STAI6: Upset”, and “STAI11: [Not] self‐confident” had the highest bridge out‐prediction (100 * bridge in‐prediction = 0.21, 0.13, 0.13, and 0.12). All the above‐mentioned nodes were among the nodes with strongest bridge centrality in bridge in‐strength or bridge out‐strength. See Figures S7 and S8 for more details about the (bridge) centrality of all nodes of the CLPN.

## 4 Discussion

To the best of our knowledge, this is the first study that conducted a longitudinal study and assessed the causal relationships between depression and anxiety in Chinese college students (*N* = 705) from the COVID‐19 lockdown period (T1) to the lockdown lift period (T2). This exploration and observation are particularly important, as depression and anxiety increased by a massive 25% in response to the emergence of this pandemic (World Health Organization 2022). Results from these analyses highlight the key bridges linking depression and anxiety, as well as key symptoms that predict the presence of mental health issues by stressful events such as social lockdown, providing a deeper understanding of how individual symptoms of depression and anxiety were interconnected, as well as the causes and causal relationships underlying the comorbidity between depression and anxiety.

Consistent with previous studies, anxiety and depression were significantly decreased after lifting the lockdown (Essadek et al. 2022; Richter et al. 2021). This may be because, after the lifting of lockdown measures, they are able to return to a more suitable study routine, more frequent physical activities, and closer social interactions, as the rigidity and time length of restrictions are triggers in terms of physical activity problems, learning mode maladaptation, social isolation, and mental health issues (Huremović 2019; Yang et al. 2023). However, there was still a considerable proportion of students exhibiting mental problems during the lockdown lift period. A previous study revealed that after returning to campus, students in the Netherlands continued to experience low to moderate levels of anxiety, depression, stress, and loneliness during the COVID‐19 pandemic (Karnbach et al. 2024). A review has suggested that although there was a light decrease in mental health problems after the easing of the lockdown restrictions, it did not reach pre‐pandemic levels in the general population (Richter et al. 2021). Growing evidence suggests that the COVID‐19 pandemic could have long‐term effects on mental health (Ahmed et al. 2021; Rahmati et al. 2023), especially among females, individuals with more severe illness status, and those with higher inflammation (Rahmati et al. 2023). Most of our participants were female, had experienced COVID‐19 infections, with the risk of recurrent infections persisting during the lockdown lift period, potentially making them more vulnerable to the long‐term impact of the pandemic. These results suggest that long‐term preventive measures against the pandemic and mental health services for emotional recovery are crucial for college students even after lifting the lockdown.

Regarding the contemporaneous networks of anxiety‐depression symptoms at T1 and T2, both contemporaneous networks exhibited extensive correlations between anxiety and depression symptoms, suggesting high comorbidity of anxiety and depression at T1 and T2. “STAI16: [Not] content” was the most central symptom and all the top four strongest symptoms existed in the anxiety community at T1. For the relationship between depression and anxiety, the strongest link connecting different communities was between “STAI17: Worried” and “BDI1: Sadness” at T1, which also had the highest‐ranking centrality in depression‐anxiety networks according to a systematic review (Cai et al. 2024). In our study, “BDI1: Sadness” and “STAI17: Worried” were also found to be the key bridge symptoms linking depression and anxiety at T1. Sadness is a main symptom of major depressive disorder outlined in diagnostic manuals (American Psychiatric Association 2013), and is influenced by stressful life events, such as the death of a loved one, health‐related events and low social support (Marroquín 2011; Slavich and Irwin 2014). Grief‐focused interventions (e.g., unguided web‐based grief intervention) demonstrate potential effects in alleviating anxiety and depressive symptoms during the COVID‐19 pandemic (Dominguez‐Rodriguez et al. 2023). On the other hand, worry is a central diagnostic feature of generalized anxiety disorder (American Psychiatric Association 2013). During the COVID‐19 pandemic, worry about family members becoming infected heightened the risk of depression, while worry about their own infection risk heightened the risk of anxiety in Chinese college students (Y. Li et al. 2021). Therefore, specific attention to mental health should be directed toward individuals living in communities with confirmed cases and those reporting worries about their families or themselves (Xiang et al. 2020). The transdiagnostic connection between the core symptoms of anxiety and depression suggests that it may serve as an important pathway contributing to their comorbidity.

There are differences in the central symptoms between the two periods. At T2, “STAI15: [Not] relaxed” took precedence as the most central symptom, and “BDI20: Worried about health” became one of the top four strongest symptoms, indicating that the importance of depressive symptoms in the network increased at T2. In addition, the transdiagnostic edge changed to that between “STAI14: Indecisive” and “BDI13: Indecisiveness”. These two symptoms were also found to be the key bridge symptoms within the top four strongest bridge nodes. Changes in lockdown policies have impacted college students' daily lives, increasing their exposure to the virus and heightening uncertainties in their daily routines, which might lead to difficulty in making decisions under uncertain time and situations (Appel et al. 2024; Appel and Gerlach 2025). These results highlight the importance of managing uncertainty arising from the pandemic. A previous study suggested that the intervention targeting intolerance of uncertainty, such as one cognitive behavioral therapy protocol “Making Friends with Uncertainty” focusing on addressing perceived and over‐estimation of threat, represents a transdiagnostic approach to alleviate depression or anxiety (Mofrad et al. 2020). It should be noted that, consistent with previous findings, the strongest links of the whole network were confined within each respective mental disorder and were identical at T1 and T2, rather than being between anxiety and depressive symptoms (Bai, Cai, et al. 2021; Bai, Xi, et al. 2021; Cai et al. 2024). These results suggest that although anxiety and depression show extensive connections and overlaps in symptoms and their interaction model may change over time, they remain distinct symptoms with stable internal structures (Borsboom and Cramer 2013).

There are novel findings about causal relationships explored by the CLPN spanning the two specific pandemic points, providing a chance to deepen understanding of the comorbidity of psychopathology. The CLPN exhibited extensive positive predictions, and the strongest edge were from “STAI13: Jittery” to “STAI7: Presently worrying over misfortunes”, and to “STAI18: Confused”, and the strongest transdiagnostic edge was from “BDI6: Punishment” to “STAI9: Frightened”. During the lockdown period, several emergency orders imposed criminal penalties for violating lockdown measures worldwide (Sun et al. 2022). College students were confined to their campuses, and leaving without permission resulted in disciplinary actions, which may have fostered feelings of punishment. After the lockdown was lifted, an outbreak occurred, potentially intensifying fright among these individuals due to their prior dissatisfaction with the lockdown policies. Although these two symptoms had relatively low in/out‐prediction or centrality, and were located at the periphery of the network, the close causal relationship between them might play a key role in pathological mechanisms of specific symptom clusters and comorbidity (Borsboom and Cramer 2013).

For predictiveness and predictability of symptoms in CLPN, “BDI15: Loss of energy” and “STAI15: [Not] relaxed” were the key symptoms in CLPN with both highest in‐prediction and out‐prediction. Loss of energy is closely associated with the concept of “Fatigue” (Chaudhuri and Behan 2004). “Fatigue” has been identified as a stable central and/or bridge symptom among Chinese college students across pandemic stages (Bai, Cai, et al. 2021; Bai, Xi, et al. 2021; Li et al. 2024), and was significantly negative related with quality of life (Jin et al. 2022). The sudden shift to a sedentary lifestyle during lockdown significantly decreased outdoor extracurricular activity (Yang et al. 2023), which might have resulted in the prevalence of fatigue and loss of energy among students in the late stage of the COVID‐19 outbreak (Liu et al. 2021). Meanwhile, as previous studies on depression‐anxiety network have showed, “Trouble relaxing” was the bridge symptoms among Chinese participants during the COVID‐19 outbreak (Wang et al. 2021), and the central symptoms among college students (Bai, Cai, et al. 2021; Bai, Xi, et al. 2021), as well as among clinicians in public hospitals (Jin et al. 2022), during the late stage of the pandemic outbreak. Our findings suggest that interventions targeting these two symptoms, such as extracurricular physical activity programs (Yang et al. 2023) and relaxation practice, should be strengthened to alleviate depression and anxiety by influencing the interactions within the entire depression‐anxiety network.

The bridge symptoms in the contemporaneous networks and in CLPN included some overlapping symptoms, such as “STAI11: [Not] self‐confident” and “STAI14: Indecisive”. College students have to deal with the stressful tasks of separation from their family and attend to numerous work and family responsibilities, making them more likely to experience mental health problems (Pedrelli et al. 2015). As mentioned above, the altered lockdown policies have increased uncertainties and may lead to feelings of indecisiveness among college students. In addition, the vast majority of our participants are medical students, who have to face heavier academic pressures and greater demands for clinical skills training. Due to the lockdown, they faced difficulties in conducting hands‐on experimental practices, and opportunities for medical residency training were also reduced. Previous studies also revealed that the changed teaching approach to online lessons and remote instruction, particularly linked to limited interaction with peers and teachers, as well as shifts in teaching methods, have undermined self‐confidence in undergraduate students and dental students (Guppy et al. 2023; Ilić et al. 2021). These crucial bridge nodes, both in terms of contemporaneous and causal relationships, may be influenced by teaching methods and the uncertainties arising from altered lockdown policies, thus influencing depression and anxiety across the pandemic period.

The 20 state‐anxiety symptoms of STAI and 21 depression symptoms of BDI were modelled in our study, unlike majority of previous depression‐anxiety network analyses predominantly using the 9 items Patient Health Questionnaire and 7‐items Generalized Anxiety Disorder scale (Cai et al. 2024). The networks with more individual symptoms may be able to provide more precise information on the relationships between symptoms, which further complements the findings of previous research. However, there are several limitations of this study. First, the study was conducted among college students in China, which may limit the generalizability of its findings to broader populations or regions worldwide. Second, depressive and anxiety symptoms were assessed using self‐report scales, which introduce the potential for biases related to recall accuracy and social desirability and affect the reliability of the results. Third, the centrality indexes used as measures have limitations. Strength provides information on the direct connections (partial correlations) between a node and its neighboring nodes, but it does not account for indirect relationships between nodes (Bringmann et al. 2019).

## 5 Conclusion

In summary, the two contemporaneous networks and the CLPN provide new insights into the internal relationships between depression and anxiety during the lockdown period and the lockdown lift period. Our results emphasize the close connection and causal relationship, as well as key bridge symptoms, in understanding the pathways between anxiety and depression, shedding light on their comorbidity and mutual influence. Although COVID‐19 is no longer classified as a public health emergency, our study provides an important theoretical basis for future preventive measures against global health crises that may lead to mental health problems among college students, and also provides further insight into minimizing the negative impacts of lockdown measures in future emergencies.

## Conflicts of Interest

The authors declare no conflicts of interest.

## Supporting information


**Table S1.** The item of State subscale of State–Trait Anxiety Inventory and Beck Depression Inventory.
**Figure S1.** Bootstrapped 95% confidence intervals of edge weights of the temporal networks at T1 and T2.
**Figure S2.** Stability of centrality indexes of the contemporaneous networks at T1 and at T2 by case dropping subset bootstrap.
**Figure S3.** Bootstrapped difference tests between node strength centrality of the 41 nodes of the contemporaneous networks at T1 and at T2.
**Figure S4.** Edge weight difference tests of the contemporaneous networks at T1 and at T2.
**Figure S5.** Stability of centrality indexes of the CLPN by case dropping subset bootstrap.
**Figure S6.** Bootstrapped difference tests between of centrality the CLPN networks at T1 and at T2.
**Figure S7.** Centrality indexes of nodes in the CLPN.
**Figure S8.** Bridge centrality indexes of nodes in the CLPN.
