# Supplementary material for: Anxiety and Depression Among College Students Before and After the COVID‐19 Pandemic Lockdown Lift: A Network Analysis Study Focus on the Transition Period
Source: Psych J. 2025 Jun 26;14(4):523–33. doi: 10.1002/pchj.70028 (PMC12318590; doi:10.1002/pchj.70028)
Supplement: Supplementary file 1 — Table S1. The item of State subscale of State–Trait Anxiety Inventory and Beck Depression Inventory. Figure S1. Bootstrapped 95% confidence intervals of edge weights of the temporal networks at T1 and T2. Figure S2. Stability of centrality indexes of the contemporaneous networks at T1 and at T2 by case dropping subset bootstrap. Figure S3. Bootstrapped difference tests between node strength centrality of the 41 nodes of the contemporaneous networks at T1 and at T2. Figure S4. Edge weight difference tests of the contemporaneous networks at T1 and at T2. Figure S5. Stability of centrality indexes of the CLPN by case dropping subset bootstrap. Figure S6. Bootstrapped difference tests between of centrality the CLPN networks at T1 and at T2. Figure S7. Centrality indexes of nodes in the CLPN. Figure S8. Bridge centrality indexes of nodes in the CLPN. [file PCHJ-14-523-s001.docx]

**Supplementary Materials**

**Anxiety and Depression Among College Students Before and After the COVID-19 Pandemic Lockdown Lift: A Network Analysis Study Focus on the Transition Period**

Jia-li Liu^1#^, Wan-ting Ran^2#^, Zhi Wang^1,2#^, Ze-min Nie^2^, Gui-lin Huang^2^, Jun-wen Yi^2^, Si-yu Yang^2^, Zi-yi He^2^, Ya Wang^3,4,5*^, Gui-fang Chen^1*^

^1^ Affiliated Hospital of Zunyi Medical University, Zuiyi, China

^2^ Zunyi Medical University, Zunyi, China

^3^ School of Psychology, Capital Normal University, Beijing, China

^4^ Neuropsychology and Applied Cognitive Neuroscience Laboratory, CAS Key Laboratory of Mental Health, Institute of Psychology, Beijing, China

^5^ Department of Psychology, University of Chinese Academy of Sciences, Beijing, China

# These authors contributed equally to this work.

* Correspondence should be addressed to Gui-fang Chen, Affiliated Hospital of Zunyi Medical University, 149 Dalian Road, Zunyi, Guizhou 563003, China. Email: [121306555@qq.com](mailto:121306555@qq.com), [chenguifang11@163.com](mailto:chenguifang11@163.com)

Or

Ya Wang, 23A Baiduizi, Haidian District, Beijing, China, Email: wangyazsu@gmail.com, wangya@cnu.edu.cn

# S1. Supplementary Tables

## Table S1 The item of State subscale of State-Trait Anxiety Inventory and Beck Depression Inventory

| STAI | T1 | T2 | BDI | T1 | T2 |
| --- | --- | --- | --- | --- | --- |
| 1. I feel calm ^[R]^ | 2.07 (0.72) | 2.05 (0.71) | 1. Sadness | 0.89 (0.55) | 0.86 (0.52) |
| 2. I feel secure ^[R]^ | 1.91 (0.75) | 1.84 (0.74) | 2. Pessimism | 0.71 (0.71) | 0.70 (0.67) |
| 3.I am tense | 1.98 (0.75) | 1.92 (0.74) | 3. Past failure | 0.79 (0.68) | 0.75 (0.69) |
| 4. I feel strained | 1.80 (0.75) | 1.74 (0.72) | 4. Loss of pleasure | 0.74 (0.58) | 0.73 (0.57) |
| 5.I feel at ease ^[R]^ | 2.23 (0.73) | 2.21 (0.76) | 5. Guilty feelings | 0.63 (0.63) | 0.55 (0.62) |
| 6.I feel upset | 2.01 (0.75) | 1.91 (0.72) | 6. Punishment feelings | 0.62 (0.68) | 0.53 (0.67) |
| 7. I am presently worrying  over possible misfortunes | 1.68 (0.79) | 1.60 (0.76) | 7. Self-dislike | 0.81 (0.57) | 0.78 (0.55) |
| 8. I feel satisfied ^[R]^ | 2.25 (0.75) | 2.26 (0.74) | 8. Self-criticalness | 0.71 (0.68) | 0.69 (0.69) |
| 9. I feel frightened | 1.70 (0.75) | 1.58 (0.70) | 9. Suicidal thoughts or wishes | 0.39 (0.56) | 0.39 (0.61) |
| 10. I feel comfortable ^[R]^ | 2.19 (0.74) | 2.17 (0.71) | 10. Crying | 0.66 (1.04) | 0.61 (1.03) |
| 11. I feel self-confident ^[R]^ | 2.29 (0.68) | 2.29 (0.71) | 11. Irritability | 0.46 (0.84) | 0.44 (0.88) |
| 12. I feel nervous | 1.75 (0.83) | 1.66 (0.81) | 12. Loss of interest | 0.67 (0.72) | 0.61 (0.70) |
| 13. I am jittery | 1.58 (0.78) | 1.46 (0.67) | 13. Indecisiveness | 0.45 (0.70) | 0.44 (0.69) |
| 14. I feel indecisive | 2.33 (0.87) | 2.30 (0.88) | 14. Worthlessness | 0.51 (0.84) | 0.47 (0.82) |
| 15. I am relaxed ^[R]^ | 2.24 (0.74) | 2.25 (0.76) | 15. Loss of energy | 0.62 (0.80) | 0.57 (0.77) |
| 16. I feel content ^[R]^ | 2.24 (0.74) | 2.25 (0.74) | 16. Changes in sleeping pattern | 0.36 (0.66) | 0.39 (0.65) |
| 17. I am worried | 2.04 (0.77) | 1.93 (0.71) | 17. Tiredness or Fatigue | 0.53 (0.71) | 0.51 (0.67) |
| 18. I feel confused | 1.84 (0.75) | 1.76 (0.75) | 18. Changes in appetite | 0.36 (0.65) | 0.35 (0.58) |
| 19. I feel steady ^[R]^ | 2.25 (0.70) | 2.29 (0.70) | 19. Lost much weight | 0.26 (0.59) | 0.25 (0.58) |
| 20. I feel pleasant ^[R]^ | 2.11 (0.72) | 2.15 (0.73) | 20. Agitation or worried about my physical problems | 0.44 (0.67) | 0.41 (0.65) |
|  |  |  | 21. Loss of interest in sex | 0.43 (0.73) | 0.37 (0.69) |

**Note.** ^[R]^ Items with reverse scoring (5 – raw score). State-STAI = State Subscale of State-Trait Anxiety Inventory; BDI Beck Depression Inventory.

# S2. Supplementary Figures


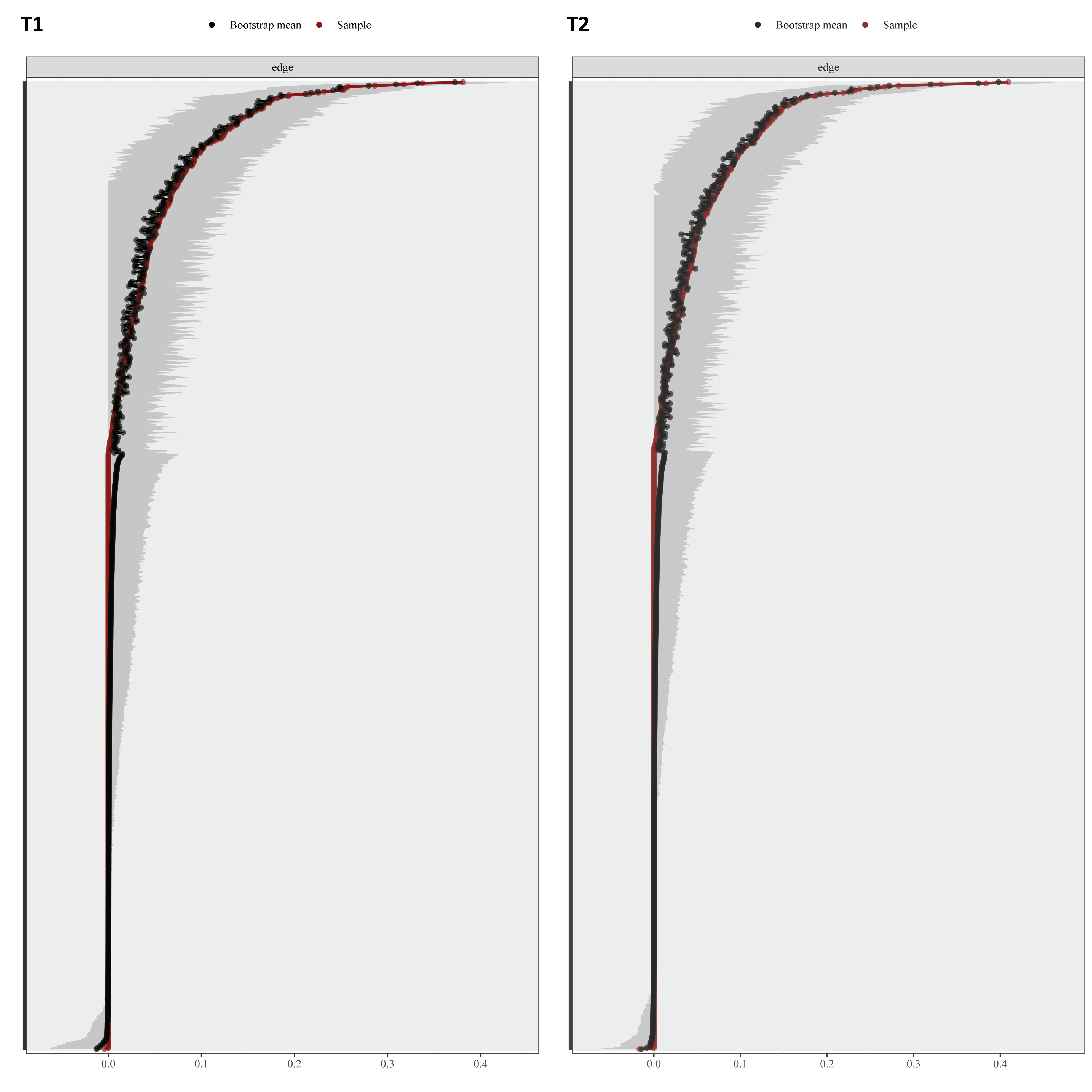


## Figure S1. Bootstrapped 95% confidence intervals of edge weights of the temporal networks at T1 and T2.

**Note.** The red dots indicate the values of each edge weight, ordered from the highest to the lowest values. The gray area represents the 95% Confidence Intervals of edge weights, estimated with the non-parametric bootstrap procedure. Wide intervals indicate lower stability and narrow intervals indicate higher stability.


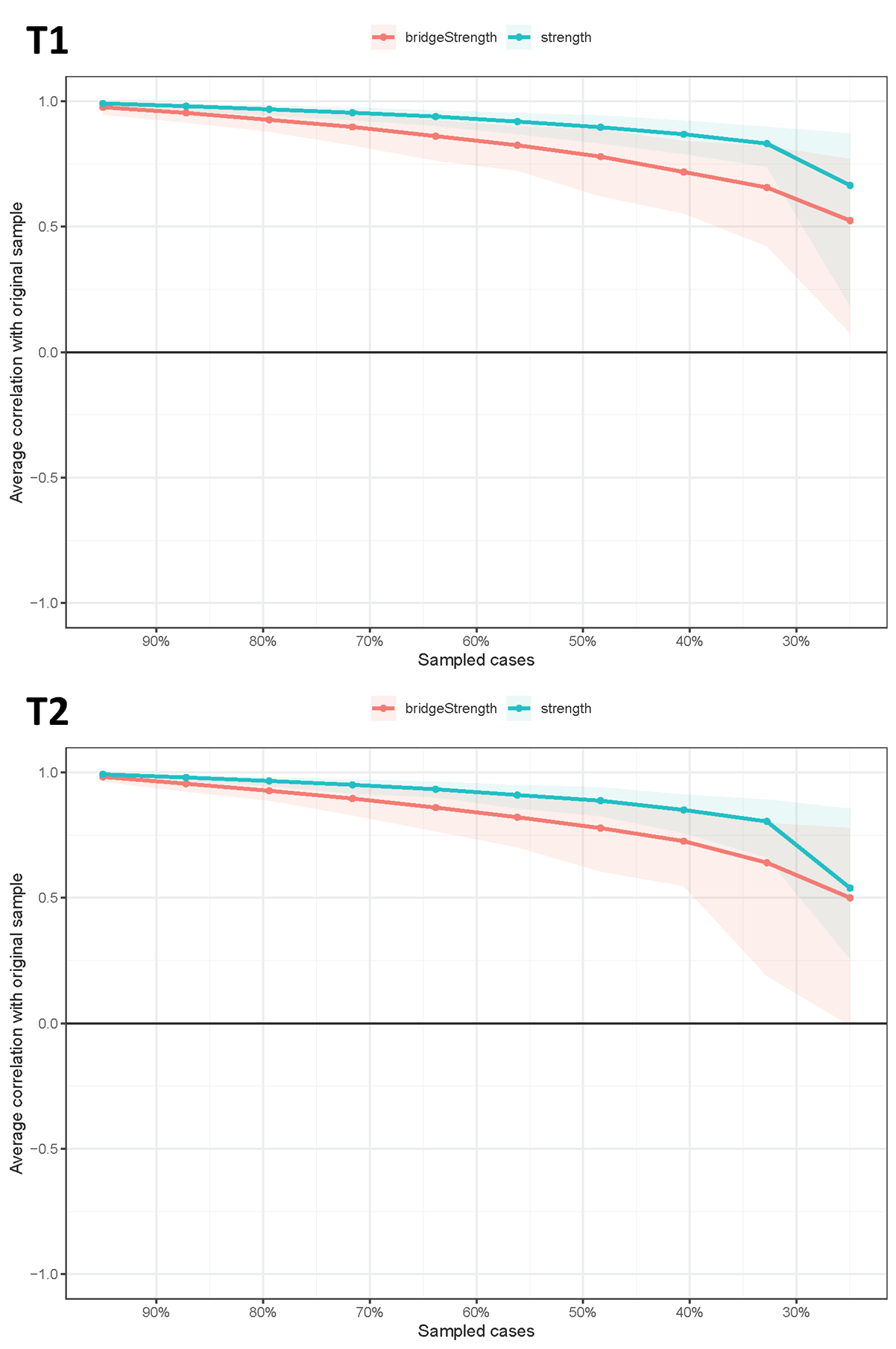


## Figure S2. Stability of centrality indexes of the contemporaneous networks at T1 and at T2 by case dropping subset bootstrap.

**Note.** The x-axis represents the percentage of cases of original sample used at each step. The y-axis represents the average of correlations between the centrality indexes from the original network and the centrality indexes from the networks of subsamples with participants dropped. Different lines indicate the correlations of strength and bridge strength while areas indicate range from the 2.5th quantile to the 97.5th quantile.


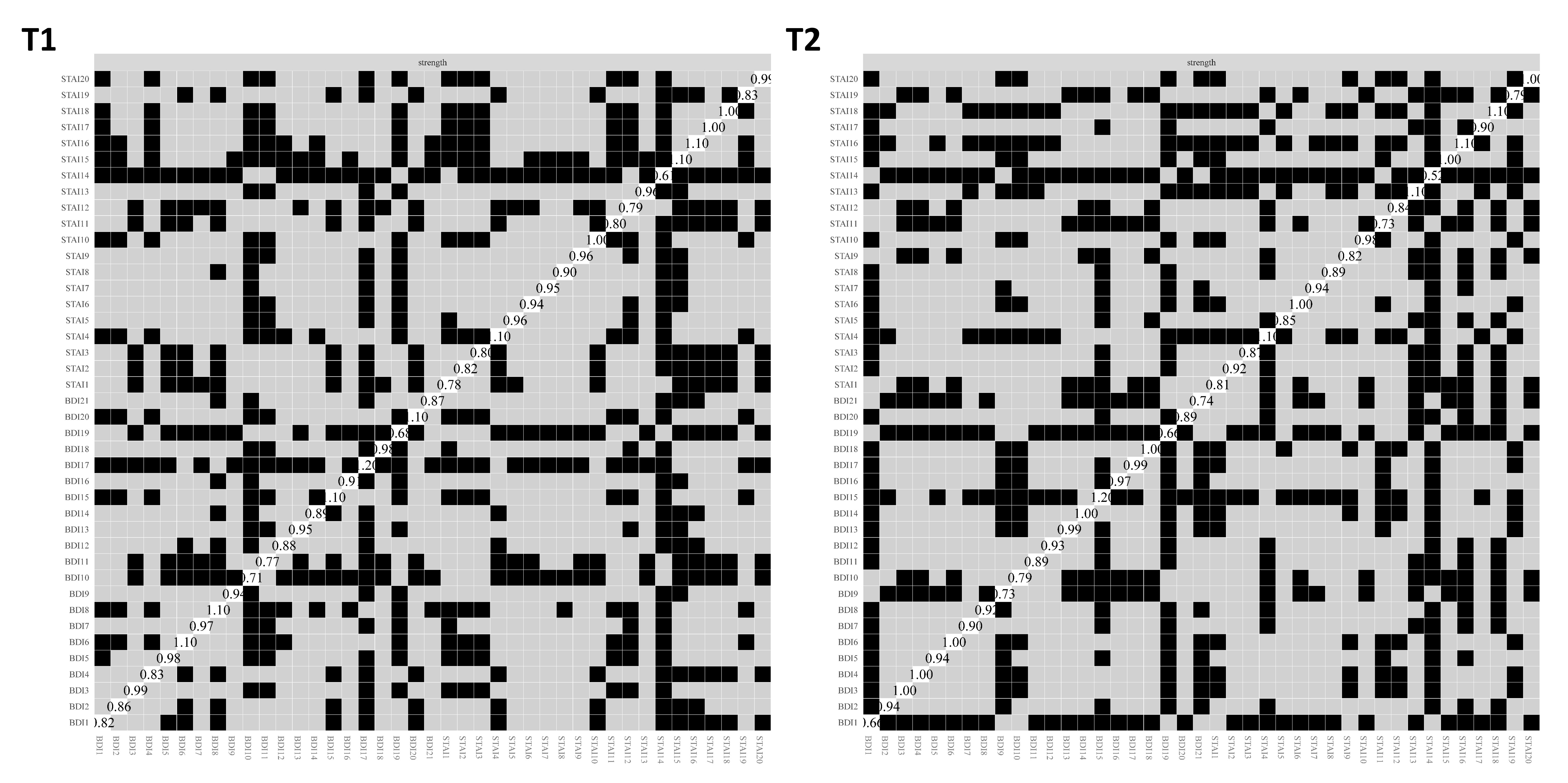


## Figure S3. Bootstrapped difference tests between node strength centrality of the 41 nodes of the contemporaneous networks at T1 and at T2.

**Note.** Black box represents a node that differs significantly from another one in strength (*p* < 0.05), and grey boxes indicate no significant difference. White boxes represent the values of node strength.

## Figure S4. Edge weight difference tests of the contemporaneous networks at T1 and at T2.

**Note.** Black boxes indicate edges that were significantly different from one another (*p* < 0.05), and grey boxes indicate no significant difference.


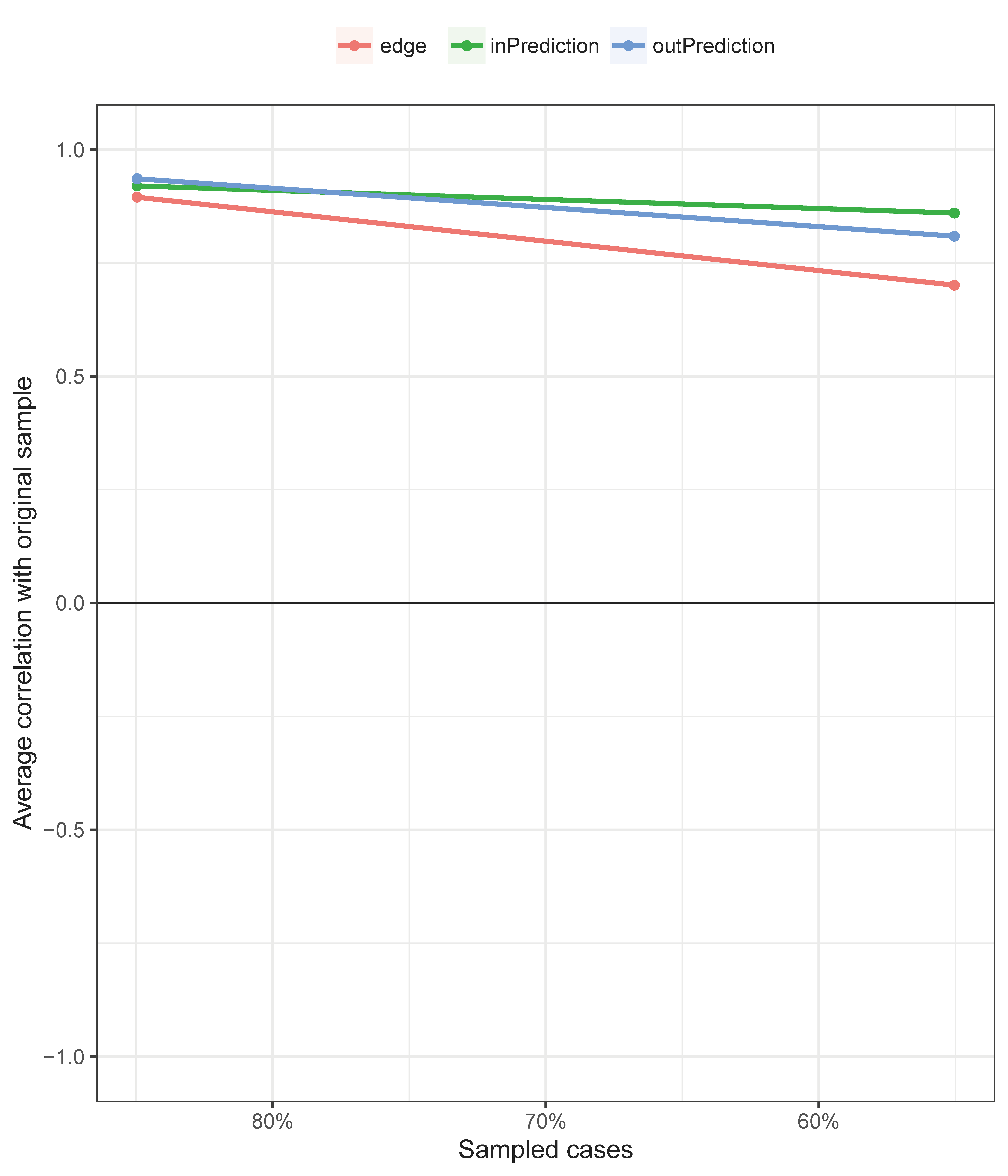


## Figure S5. Stability of centrality indexes of the CLPN by case dropping subset bootstrap.

**Note.** The x-axis represents the percentage of cases of original sample used at each step. The y-axis represents the average of correlations between the centrality indexes from the original network and the centrality indexes from the networks of subsamples with participants dropped. Different lines indicate the correlations of edge, in-strength, and out-strength.


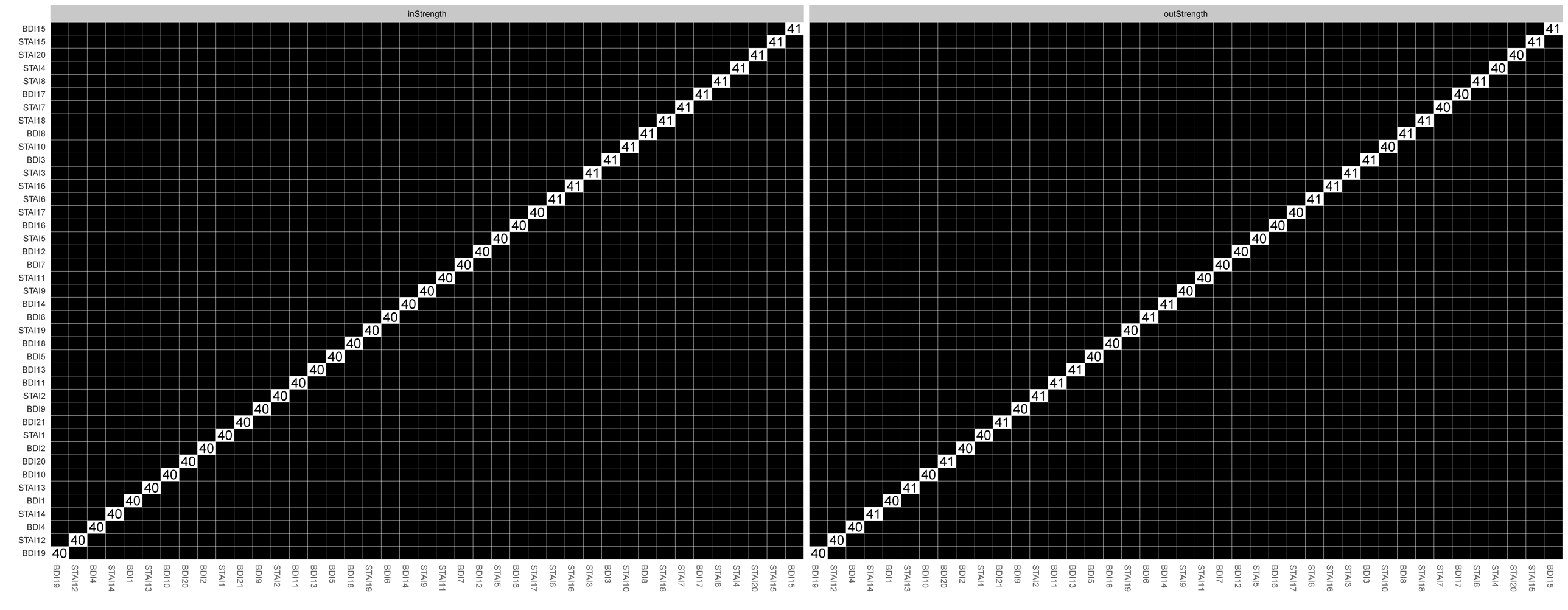


## Figure S6. Bootstrapped difference tests between of centrality the CLPN networks at T1 and at T2.

**Note.** Black box represents a node that differs significantly from another one in strength (*p* < 0.05), and grey boxes indicate no significant difference.


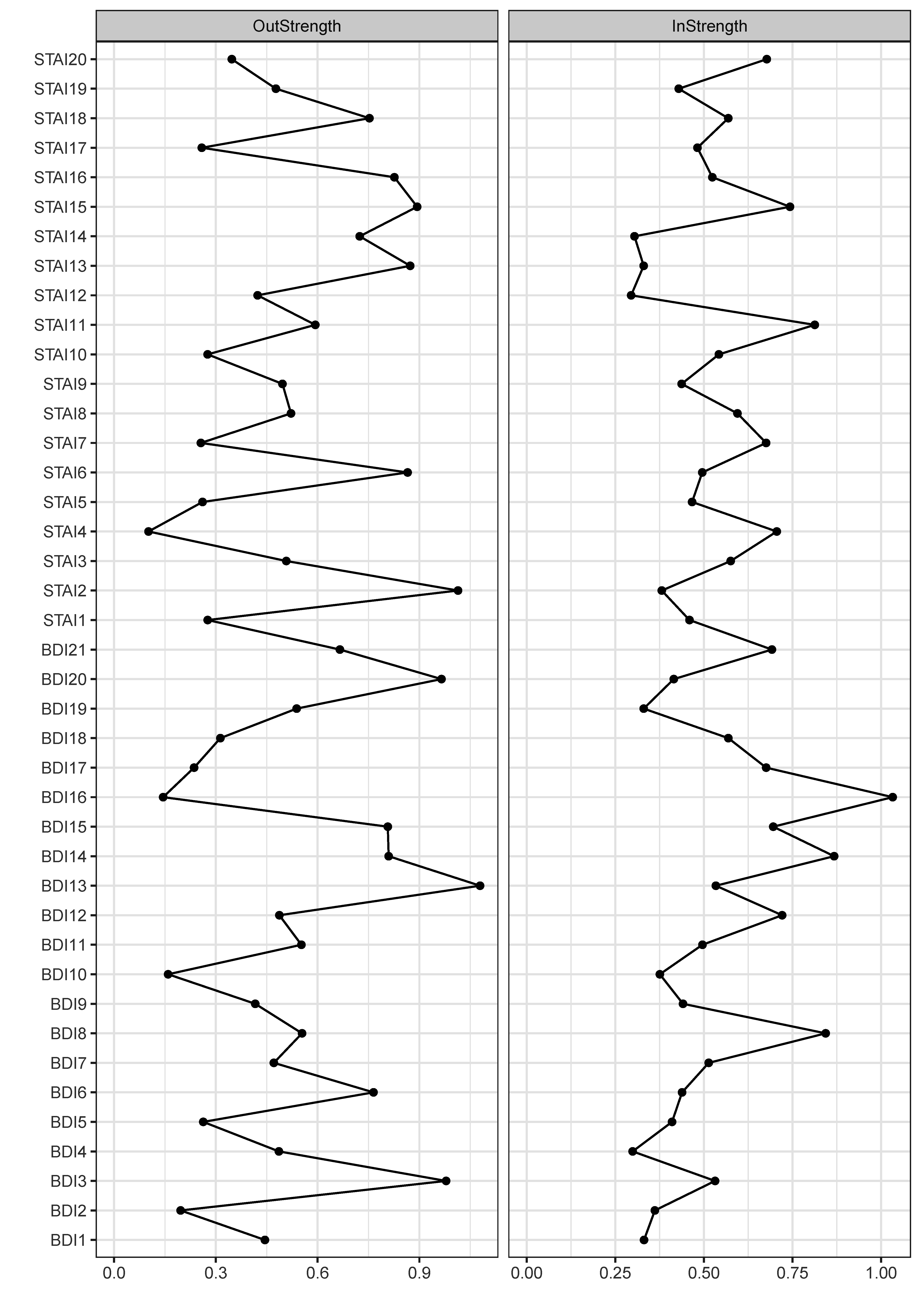


## Figure S7. Centrality indexes of nodes in the CLPN.


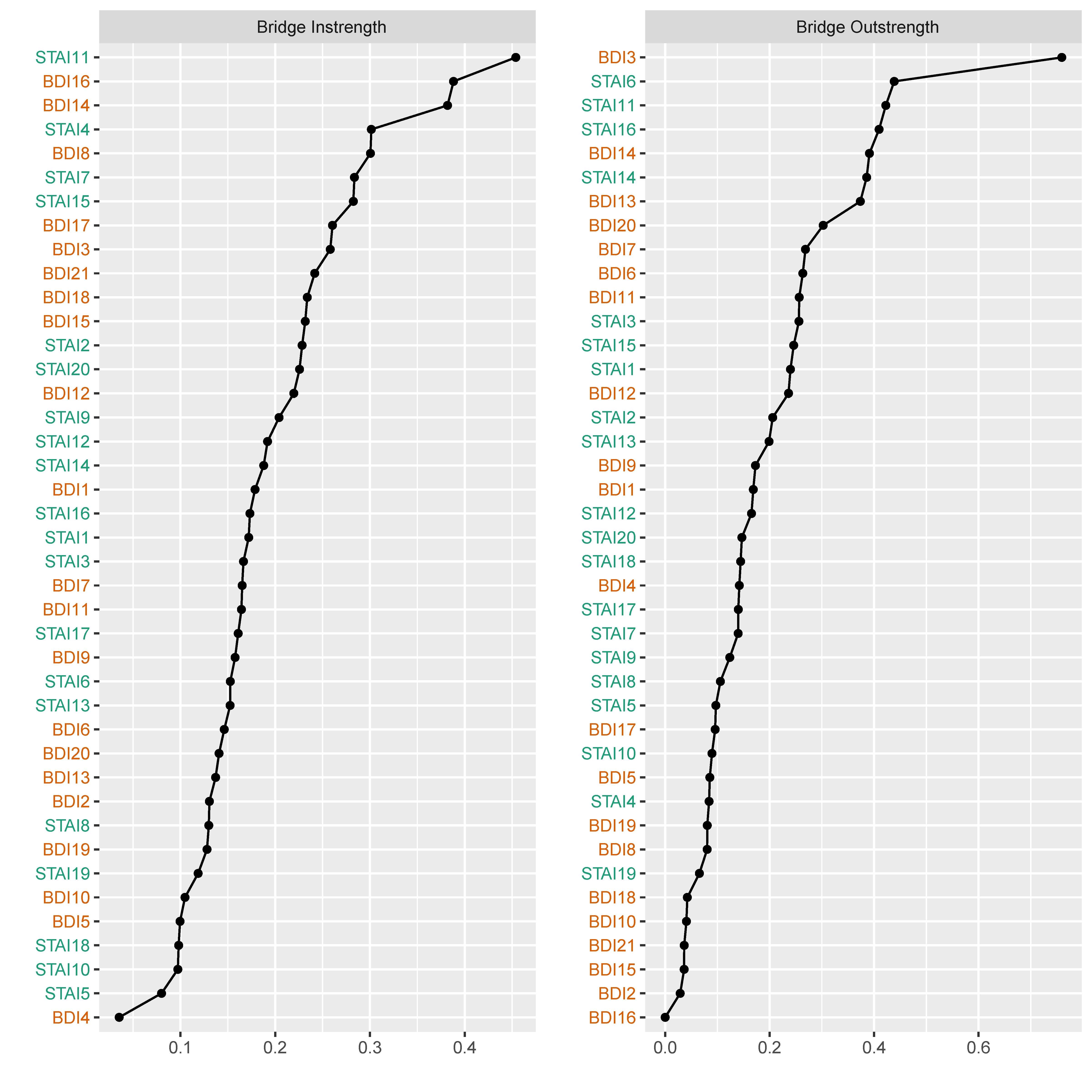


**Figure S8. Bridge centrality indexes of nodes in the CLPN.**
